# Progress in drug delivery and diagnostic applications of carbon dots: a systematic review

**DOI:** 10.3389/fchem.2023.1227843

**Published:** 2023-07-14

**Authors:** Hemlata Kaurav, Dhriti Verma, Amit Bansal, Deepak N. Kapoor, Sandeep Sheth

**Affiliations:** ^1^ School of Pharmaceutical Sciences, Shoolini University of Biotechnology and Management Sciences, Solan, Himachal Pradesh, India; ^2^ Formulation Research and Development, Perrigo Company Plc, Allegan, MI, United States; ^3^ Department of Pharmaceutical Sciences, College of Pharmacy, Larkin University, Miami, FL, United States

**Keywords:** carbon dots, drug delivery, gene delivery, nanocarriers, bioimaging

## Abstract

Carbon dots (CDs), which have particle size of less than 10 nm, are carbon-based nanomaterials that are used in a wide range of applications in the area of novel drug delivery in cancer, ocular diseases, infectious diseases, and brain disorders. CDs are biocompatible, eco-friendly, easy to synthesize, and less toxic with excellent chemical inertness, which makes them very good nanocarrier system to deliver multi-functional drugs effectively. A huge number of researchers worldwide are working on CDs-based drug delivery systems to evaluate their versatility and efficacy in the field of pharmaceuticals. As a result, there is a tremendous increase in our understanding of the physicochemical properties, diagnostic and drug delivery aspects of CDs, which consequently has led us to design and develop CDs-based theranostic system for the treatment of multiple disorders. In this review, we aim to summarize the advances in application of CDs as nanocarrier including gene delivery, vaccine delivery and antiviral delivery, that has been carried out in the last 5 years.

## 1 Introduction

**FIGURE 1:**
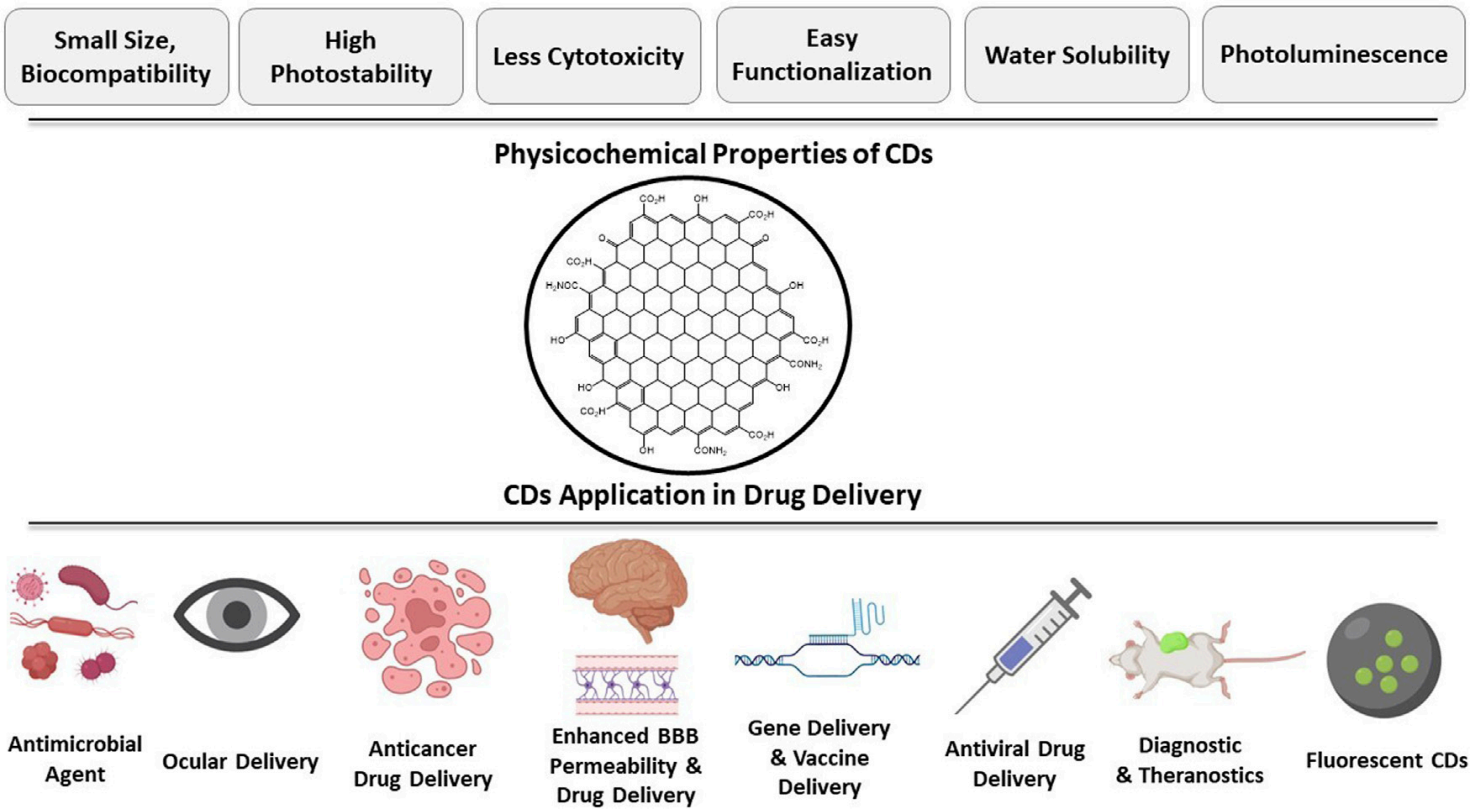
Applications of CDs in therapeutic drug delivery.

First discovered by Walter Scrivens and his colleagues in 2004 (Xu et al., 2004), carbon dots (CDs) possess properties of good photostability, biocompatibility, adaptability, less cytotoxicity, high chemical inertness, easy method of synthesis, eco-friendliness, easy functionalization, non-blinking photoluminescence, and better water solubility. These properties make CDs suitable for use in various fields like drug delivery, bioimaging, and optoelectronics (Ren et al., 2019). In pharmaceuticals, CDs are mainly used as a nanomaterial for drug delivery in various disorders such as cancer, neurological disorders, eye diseases, infectious diseases and for gene delivery (Figure 1) (Zhang et al., 2019a). CDs have functional groups on their surfaces to enhance their water solubility and allow conjugation with organic, inorganic polymers and biomolecules for different applications (Singh et al., 2017). Functional groups, specifically amino, carboxyl, and hydroxyl groups on CDs surface also help further modifications that improve their biocompatibility, optical properties, target ability while increasing their sensitivity and selectivity (Zuo et al., 2016).

The application of CDs depends upon their interaction with the analytes. The interaction of analyte with CDs either decreases fluorescence by quenching or increases fluorescence by inhibiting the quenching effect. The CDs quenching mechanism consists of static quenching, energy transfer, dynamic quenching, photoinduced electron transferred, and inner filter effect. Energy transfer further includes dexter energy transfer, fluorescence resonance energy transfer, and static energy transfer (Zu et al., 2017). CDs gives the advantage to choose surface functionality through electrostatic interaction for selective drug molecules (Singh et al., 2017). To date, different types of CDs have been synthesized for multiple purposes such as for therapeutic drug delivery, drug targeting, gene delivery, biosensing, chemical sensing, bioimaging electrolysis, etc. Researchers also discovered that under near infrared (NIR) light stimulation, CDs emit in the NIR spectral range, expanding its applicability in drug administration, bioimaging, photoacoustic imaging, and anticancer treatment. Due to the excellent photostability and fluorescent property, CDs can also be used as probes in analytical field (Zuo et al., 2016). Recently, green and red-emitting CDs are synthesized to characterize the normal cell and cancer cell through bioimaging (Zeng et al., 2016; An et al., 2021).

Reportedly, research shows that CDs can suppress growth of HepG2 human hepatocellular carcinoma cells (Li et al., 2014). It can also suppress breast cancer cells by generating a huge amount of reactive oxygen species (Hsu et al., 2013). CDs can pass the blood-brain barrier (BBB), which helps in treating brain-related diseases like Alzheimer’s and brain tumor (Dong, 2018; Zhang W. et al., 2021). Worldwide, a significant number of researchers are working on CDs-based drug delivery systems to evaluate their versatility and efficacy in the field of pharmaceuticals. As per the reported scientific data, there is a tremendous increase in our understanding of the physicochemical properties, diagnostic and drug delivery aspects of CDs, which has helped researchers to design and develop CDs-based theranostic system for the treatment of multiple disorders. CDs has potential application in antimicrobial delivery, gene delivery, ocular delivery, cancer targeted delivery, vaccine delivery, antiviral delivery, drug targeting to neurological conditions and enhancing BBB penetration. In this review, we have discussed recent advances in CDs research with special emphasis on their drug delivery applications for different diseases.

## 2 Structure, method of synthesis and properties of CDs

### 2.1 Structure

Structurally, the average size of CDs is reported to be less than 10 nm and they may be either graphitic or amorphous in nature (El-Shafey, 2021). CDs exhibit dot like structure with varying size and surface morphology depending up on the selection of precursor and method of synthesis (Wang B. et al., 2022). Amorphous core shell structure with mixed sp^2^/sp^3^ hybridization is suggested by some researchers while others have suggested graphitic crystalline structure with sp^2^ carbon (Mintz et al., 2021; Yadav et al., 2023). CDs are endowed with various functional groups on their surface, which may include hydroxyl (–OH), carboxyl (–COOH), aldehyde (–CHO), amino (–NH_2_) and sulfhydryl (–SH) group. The type of functional group present on the surface is governed by the precursor used for the preparation of CDs which can further affect their properties. For example, oxygen containing functional groups imparts negative charge to CDs, whereas nitrogen containing functional groups give positive charge, and the difference in charge affects the passivation capabilities of CDs. Therefore, it is important to know the functional groups present on the surface of CDs that can be determined using various conventional techniques such as ninhydrin colorimetry for the determination of amino groups and Boehm titration for carboxyl group determination. The analytical attributes of CDs can be characterized by using transmission electron microscopy (TEM) and X-ray diffraction (XRD).

### 2.2 Synthesis of CDs

A wide range of methods are available for the preparation of CDs. An ideal method is the one that can synthesize CDs of uniform size, with high quantum yield (QY), scalable, and cost effective. CDs are generally synthesized by two methods, i.e., top-down and bottom-up.

#### 2.2.1 Top-down method

The top-down approach for synthesis of CDs involves breakdown of large carbon precursors (graphene, ash, or soot) to generate nanosized particles (Carbonaro et al., 2019). Different top-down methods include arc discharge, oxidative cracking, laser ablation and electrochemical oxidation (Lim et al., 2015). The arc discharge method of making CDs has the limitation of forming non-uniform size CDs and purification. Other methods such as laser ablation and oxidative cracking uses toxic reagents passivation and are therefore not the preferred choice. The electrochemical oxidation method uses electrolytic graphite rods that can produce crystalline CDs with high photocatalytic activity (Ming et al., 2012). Moreover, the CDs produced by electrochemical oxidation method confer protection from immune-mediated hepatitis due to the interference with activation of T cells and macrophages and their inherent accumulation in liver. The CDs produced by electrolysis have fewer surface functional groups resulting in its poor water dispersibility. This deficiency can be overcome by adding surface functional groups via refluxing.

Top-down approaches provide the advantage of scalability and availability of abundant raw materials for the synthesis of CDs. However, it suffers from the limitation of producing CDs of non-uniform morphology with a wider size distribution. It may also introduce impurities in CDs that can affect its fluorescence property (Wang B. et al., 2022).

#### 2.2.2 Bottom-up method

The bottom-up approach involves production of CDs using carbon precursors that are small organic molecules and polymers containing -OH, -COOH, and -NH_2_ functional groups. The common procedure used involves dehydration followed by carbonization resulting in formation of CDs with stable properties and uniform morphology with narrow particle size distribution. The bottom-up approach for making CDs includes synthesis via microwave, hydrothermal method, template assisted, cage opening, etc. (Wang B. et al., 2022). Among these, microwave and hydrothermal methods have gained more attraction and are discussed in more detail herein.

##### 2.2.2.1 Microwave

The microwave method used to synthesize CDs provides an efficient route due to its fast heating rate and reaction kinetics. This method produces CDs that are uniform in size and the size obtained can be controlled using microwave power and treatment time. Microwave causes the dehydration and pyrolysis of reaction precursor followed by carbonization to form CDs (Xu et al., 2016). Despite the benefits that microwave method offers, it suffers from the limitation of making unwanted byproducts and needs extensive purification process. Tables 1 and 2 shows several studies in which microwave method was used to make CDs that were later used for biomedical application.

**TABLE 1:** Recently reported CDs as antimicrobials and their applications for delivery of antimicrobials.

No.	Nanomaterial	Precursor	Method of preparation	Applications	Ref.
1	Nitrogen co-doped CDs-genipin covalent conjugate (N-CDs-GP)	Genipin	Hydrothermal Method; QY was 4.7%	Antibacterial agent, Selective Gram-positive bacterial	Chu et al. (2020)
2	Nitrogen-doped CDs (N-CDs)	Meta-phenylenediamine	Hydrothermal Method; QY was 12%	Eradication of Gram-negative and Gram-positive bacteria	Saravanan et al. (2020)
3	Nitrogen-doped CDs (N-CDs)	polyethylenimine (PEI) and citric acid (CA)	Hydrothermal Method; QY was 53%	Inhibition of bacteria and good topical delivery	Demirci et al. (2020)
4	Nanofiltration Membrane Nitrogen-doped CDs (N-CDs)	Polyethersulfone (PES)	Hydrothermal technique	Improved anti-microbial results as compared to the plain PES	Koulivand et al. (2020)
5	Photoluminescent CDs	Wheat bran conjugated with amoxicillin	Hydrothermal method; QY was 33.23%	Minimal cytotoxic, Increased Drug Loading Efficiency, Drug Delivery of Amoxicillin	John et al. (2020)
6	Nitrogen, sulphur co-doped graphene quantum dots (N, S-GQDs	Octa-imine substitution of zinc phthalocyanine (ZnPc)	π-π stacking	Antimicrobial PDT	Sen and Nyokong (2021)
7	CDs	Citric acid	One-pot microwave-assisted synthesis	Photodynamic antimicrobial action, useful for treating wounds infected with Gram-positive bacteria	Romero et al. (2021)
8	Fluorescent surface-quaternized CDs (JB-CDs)	Jute caddies, benzalkonium chloride	Sonochemical method of preparation	Chemical sensing, Antibacterial Activity, Nanocarrier for ciprofloxacin delivery	Das et al. (2020)
9	Fluorescent CDs	Levofloxacin hydrochloride	Hydrothermal method	Antibacterial activity, Synergetic mechanism of CDs with Levofloxacin hydrochloride	Liang et al. (2021)
10	CDs	*Curcuma longa*	Hydrothermal method	Antibacterial activity against Gram-positive and Gram-negative bacteria	Saravanan et al. (2021)
11	Chlorhexidine gluconate CDs (CGCDs)	Chlorhexidine gluconate	Hydrothermal method	Antibacterial effect against both Gram-positive and Gram-negative bacteria	Sun et al. (2021)
12	Blue–green emitting CDs (CDs)	Glucose, citric acid, polyethylenimine	Hydrothermal method; QY was found to be 14%	Antibacterial activity against Gram-negative and Gram-positive Bacteria	Zhao et al. (2021)
13	Negative-charge CDs (CDs)	Citric acid, Urea	Microwave assisted synthesis	Antimicrobial therapy	Kung et al. (2020)
14	Fluorescent blue/green CDs	Oyster mushroom (*Pleurotus species*)	Hydrothermal method	Antibacterial activity against Gram-negative and Gram-positive Bacteria	Boobalan et al. (2020)
15	CDs	*P*-phenylenediamine	One-step hydrothermal method	Antibacterial activity for both Gram-negative and Gram-positive Bacteria	Ye et al. (2020)
16	CDs	Citric acid and β-alanine	One-pot microwave-assisted synthesis	CDs are a viable alternative to commercially supplied antibiotics	Pandey et al. (2021)
17	Xylitol CDs (XCDs)	Xylitol (XLT) conjugated with ketoconazole and tetracycline	Microwave-assisted carbonization	Good drug carrier and at low dose high inhibition was reported	Ahuja et al. (2021)
18	Levofloxacin-basedCDs (LCDs)	Levofloxacin hydrochloride	One-step hydrothermal method	Greater antibacterial activities and low drug resistance	Wu et al. (2022)

**TABLE 2:** Drug delivery application of carbon dots in treatment and diagnosis of cancer.

No.	CD type	Precursor	Method of preparation	Anticancer drug	Applications	Ref.
1	Fluorescent CDs	κ-carrageenan and folic acid	Facile hydrothermal process; quantum yield (QY) obtained was 76.12%.	Capecitabine (cap)	Can be used as nano-vehicle for anticancer drug, biomedical studies and cancer cell targeting	Das et al. (2019)
2	Fluorescent CDs	Polyethylene glycol (PEG), gelatin	Microwave carbonization; QY obtained was 34%.	Methotrexate (MTX)	CDs-PEG showed good antitumor efficacy than free MTX	Arsalani et al. (2019)
3	Mesoporous silica nanoparticles (MSNs) coated with CDs and poly-N-vinylcaprolactam (PNVCL)	Poly (N-vinylcaprolactam) (PNVCL)	Schiff base reaction	Doxorubicin (DOX)	Controlled drug release; CDs showed low toxicity and killed cancer cells without affecting normal cells	Li et al. (2020a)
4	Red-emissive carbon quantum dots (CQDs)	Nitrogen	One-step solvothermal method; QY was 20%	DOX	CQDs can be targeted to nuclei of the cancer cells and cancer stem cells	Su et al. (2020a)
5	5-ALA-CQD-Glub-CD based nanocarrier system	5-aminolevulinic acid, mono-(5-BOC-protected-glutamine-6-deoxy) β-cyclodextrin (COD-Glu-β-CD)	Hydrothermal method	DOX	Chemo/photodynamic synergistic effects on cancer therapy, ROS production causes cell damage and morphological alterations in breast MCF-7 cell line	Li et al. (2020b)
6	Fluorescent CDs	Polyethylenimine (PEI)	Microwave hydrothermal carbonization	DOX	Improved and prolonged drug release, CD-PEI-DOX showed better cytotoxic effect in liver cancer cells (MHCC-97L and Hep3B) as compared to plain DOX	Hailing et al. (2020)
7	DOX-CDs	Citrate and urea	Hydrothermal method	DOX	Biomedical imaging showed excellent aqueous stability, photoluminescence property, and a high quantum yield of 93%. Intracellular drug delivery, DOX-CDs showed cytotoxicity in ovarian cancer cell line H0-8910	Sun et al. (2020)
8	CDs	Folic acid	Hydrothermal method; QY was 97%.	DOX	Potential in biological imaging and in drug delivery, better targeting ability and stronger fluorescence intensity of FA-CDs-DOX makes it easy to penetrate tumor tissue and skin	Wang et al. (2020)
9	Nitrogen doped carbon quantum dots (CQD)	Citric acid and urea	Hydrothermal method	5-Fluorouracil (5-FU)	5-FU-CQD nanoconjugate can improve effectiveness and reduced side effects of 5-FU	Cutrim et al. (2021)
10	Fluorescent CQD	o-phenylenediamine	Synthetic microplasma liquid method	---	Good solubility, non-toxic, and high biocompatibility, photodynamic therapy for treating cancer. Yellow fluorescence can be used for marking HeLa cancer cells	Qin et al. (2021)
11	Red emissive polymer CDs	Thiophene phenylpropionic acid	Hydrothermal method	Coptisine	High drug loading efficiency (>96%), prolonged drug release and improved effectiveness against cancer cells, tumor-targeting potential and drug release tracer	Ren et al. (2021)
12	CQDs	Gallic acid (GA) used as carbon resource, folic acid was used as the nitrogen resource and citric acid monohydrate (CA) was used as the auxiliary carbon source.	Microwave-assisted method	Gallic acid	Showed good targeting imaging and antitumor abilities towards HeLa cells	Lv et al. (2021)
13	CDs	Ethylenediamine	Hydrothermal method	Curcumin (Cur)	Excellent solubility, Improved stability and enhanced bioavailability of CurCDs as compared with plain curcumin	Sharma et al. (2021)
14	CDs	Aldehyde precursors, chitosan	Solvothermal method	Chitosan nanocomp-osite hydrogels	3D nanocomposite prepared for microRNA-21 detection in MCF-7 breast cancer cells (LOD—0.03fM) showed low probe cytotoxicity	Mohammadi et al. (2021)
15	CDs	GSH-sensitive carbonyl vinyl sulfide linkage, and surface decoration with biotin	Direct conjugation	6-Mercaptopu-rine (6-MP)	Showed excellent stability, lower cellular toxicity and good activity against MCF-7 and HepG2	Talib and Mohammed (2021)
16	CDs	Folic acid	Microwave assisted method	DOX	Excellent therapeutic and diagnostic capability	Fahmi et al. (2021)
17	Nitrogen and sulphur co-doped fluorescent CDs	Methyl-β-cyclodextrin (Me-β-CD)	Hydrothermal method	Mitoxantr-one (MTO)	CDs exhibited biocompatibility and greater photobleaching which can be used in cell imaging and drug tracking	Wen et al. (2020)

##### 2.2.2.2 Hydrothermal

This is the most commonly used method for CDs synthesis as it is reported to be ecofriendly and inexpensive. This method has gained a lot of popularity and is found to surpass the benefits of microwave methods. The CDs produced by hydrothermal method are endowed with hydrophilic surface functional groups such as -OH, -COOH, or -NH_2_ groups and thus imparts good water dispersibility (Wang et al., 2019).

In the past few years, the synthetic procedures for the fabrication of fluorescent CDs have become significantly advanced. However, developing simple methods for the synthesis of multifunctional CDs with high QY is still challenging due to limited availability of raw materials and multiple variables involved in the process (Zuo et al., 2016; An et al., 2021). Recently, fabrication of CDs using plant material as a carbon source, such as orange juice (Sahu et al., 2012), beetroot extract (Singh et al., 2018), bread, jaggery and sugar (Sk et al., 2012), has been reported.

### 2.3 Properties of CDs

CDs have gained a lot of attraction in recent years due to their unique optical properties and biocompatibility. CDs possess optical properties which include absorption and photoluminescence. Due to these properties, CDs are reported to have potential application in the field of bioimaging and theranostics (Naik et al., 2022). CDs are also reported to have biocompatibility and low toxicity which allows the application of CDs as nanocarrier in the field of drug delivery and diagnostics (Biswal and Bhatia, 2021).

#### 2.3.1 Absorption

The absorption behavior of CDs depends on its method of preparation and precursor carbon source. CDs show strong absorbance in the UV region of 200–400 nm and that further extends to visible range. The absorption bands in the visible region are assigned π- π* of C=C bond or n- π* transition of C=O/C=N bond (Adrita et al., 2020; Liu et al., 2020). Some CDs that emit red or near infrared (NIR) emission possess conjugated *π* domains that allow absorption in the range of 50–800 nm. Moreover, CDs absorption properties are further affected by the size of *π* conjugated domains, type and content of surface functional groups, and the oxygen/nitrogen content of the carbon cores (Biswal and Bhatia, 2021).

#### 2.3.2 Photoluminescence

Photoluminescence is one of the most important properties of CDs finding wide range of applications especially in the field of biomedical sciences. Various parameters such as size, morphology, internal structure, and composition affect the photoluminescence of CDs. Moreover, the method of synthesis, surface passivation, and the precursor used regulates the photoluminescence of CDs. Among the methods, synthesis via hydrothermal carbonization and microwave are found to augment the photoluminescence of CDs. For passivation, polyethylene glycol (PEG) and polyethylenimine (PEI) are some of the commonly used passivating agents (Biswal and Bhatia, 2021). Studies have shown that imparting chirality to CDs results in enhanced light stability and provides long term imaging. The addition of L-cysteine to the surface of CDs forming L-CDs allows precise imaging of dynamic changes in the Golgi apparatus in the early stages of an infection (Li et al., 2017).

#### 2.3.3 Toxicity of CDs

The increased possibility of the use of CDs in humans has mandated the evaluation of its toxicity profile. The toxicity of CDs can be evaluated by *in vitro* and *in vivo* methods. For *in vitro* methods, assays such as MTT or WST-1 are frequently used in which cultured cells are exposed to CDs and cell viability is determined and compared with the positive control and naïve group (Zhang M. et al., 2021). For *in vivo* study, CDs are administered to mice or zebra fish and toxicity is assessed through blood analysis, hematological analysis, and inflammatory analysis on various organs such as kidney, spleen, liver, etc (Singh et al., 2018). The prevalence of reports on the toxicity of CDs suggests that CDs are either non-toxic or minimally toxic. The toxicity of CDs depends on their individual physicochemical properties such as surface charge, concentration, photolysis, etc. It has been found that a positive charge on CDs is mainly responsible for its toxicity. Further studies have revealed that increased surface charge density causes significant oxidative stress, IL-8 release, and mitochondrial dysfunction resulting in airway inflammation or allergen-induced immune response (Weiss et al., 2021).

One of the *in-vitro* studies used HeLa cells for toxicity evaluation of reactive red 2 (RR2), a raw material used for the synthesis of CDs. The results obtained showed that CDs prepared from RR2 showed 70% more cell viability than its precursor, RR2. The *in vivo* studies performed on zebra fish showed reduced cytotoxicity of CDs and thus further confirmed its safety at preclinical level (Chen W. et al., 2021; Wang B. et al., 2022). Furthermore, to evaluate the biological distribution of CDs in mammals, radiolabeling of CDs was done and the results revealed that CDs were eliminated via kidney and fecal excretion (Tao et al., 2012). This indicates that CDs does not get accumulated and have negligible toxicity *in vivo*.

## 3 CDs applications in drug delivery

### 3.1 CDs in antimicrobial drug delivery

**FIGURE 2:**
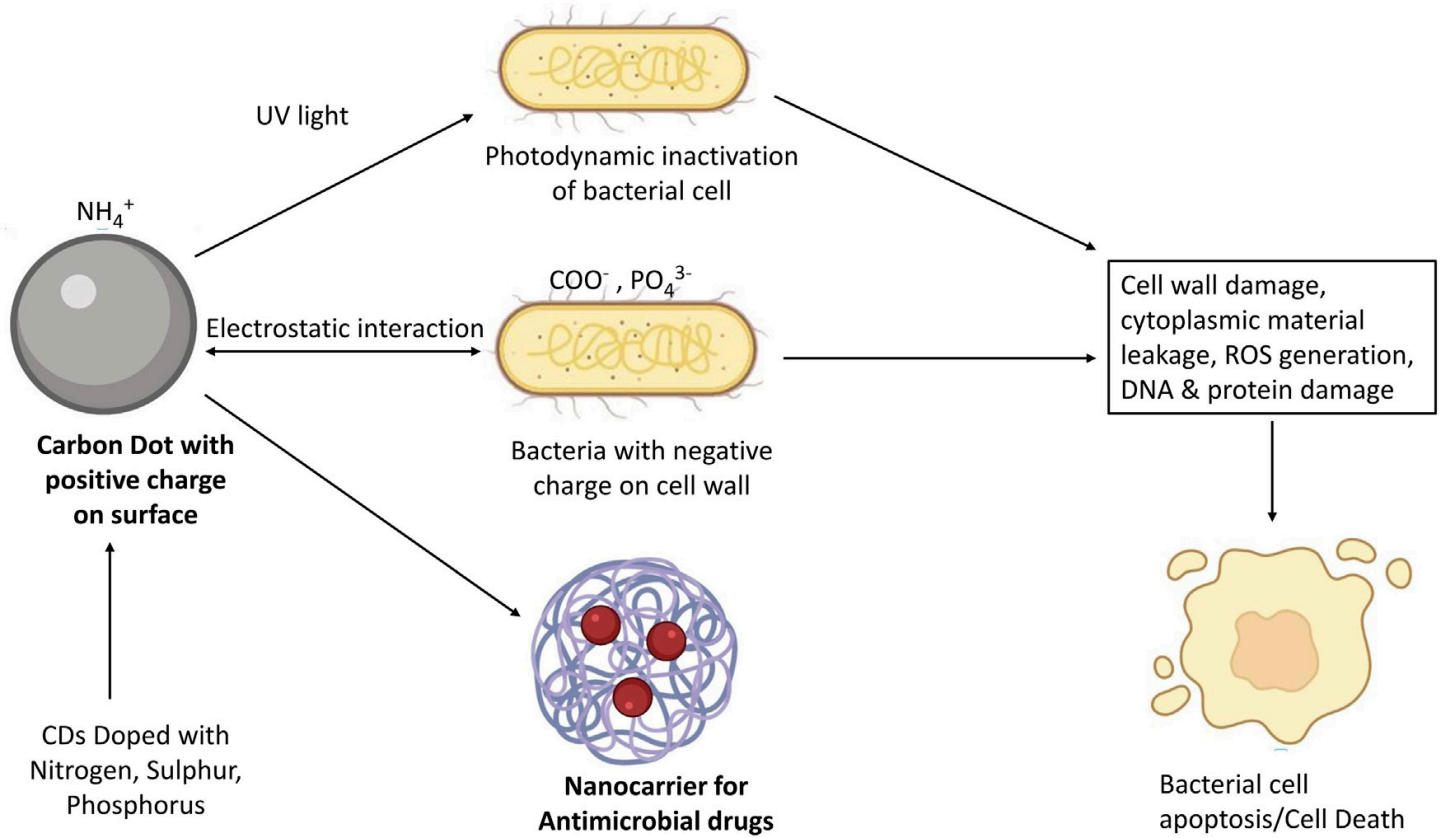
Mechanism of CDs for antimicrobial activity.

Microbial infection is caused by a number of microorganisms such as *Escherichia. coli, Salmonella typhimurium, Streptococcal pneumoniae Bacillus cereus, Mycobacterium tuberculosis, clostridium perfringens, and Staphylococcus aureus* (Cui et al., 2020). There is a wide range of antibiotics which are used against these infectious diseases. Inconsistent and regular use of antibiotics are now the main cause of developing antimicrobial resistance (AMR) among humans. Due to AMR, small injuries and common infections will become life-threatening diseases (Zhao et al., 2019). Thus, it is necessary to develop an antimicrobial substrate against resistant bacteria. CDs have significant potential for microbial imaging and itself acts as antimicrobial agents owing to their physicochemical and optical properties which includes simple method of preparation, low toxicity, excellent photostability, good water dispersibility, and flexible surface functionalization (Dong et al., 2020). Antibacterial mechanism of CDs occurs via electrostatic interaction between negatively charged bacterial cell walls and positively charged CDs. It will cause bacterial cell wall lysis and cell apoptosis. CDs acts as a photosensitizer nanomaterial for detection and photodynamic inhibition of bacterial strains, which is a one of the most prominent approaches to fight against multidrug resistance (MDR) pathogens (Figure 2) (Ghirardello et al., 2021).

#### 3.1.1 Antimicrobial applications of nitrogen-doped CDs

Antibacterial activity of CDs mainly depends upon the nitrogen (N) content and their surface charges. In this context, many studies utilized amines or quaternary ammonium salts as precursors for cationic group-based CDs probes synthesis. Also, phosphorus and sulphur-doped CDs were reported as antimicrobials (Ghirardello et al., 2021). Chu et al. (2020), produced nitrogen co-doped CDs-genipin covalent conjugate (N-CDs-GP) for Gram-positive bacterial bioimaging and as antibacterial agents. Saravanan et al. (2020), synthesized nitrogen-doped CDs (N-CDs) in which meta-phenylenediamine was used as a source of carbon and nitrogen. The N-CDs showed bright blue color fluorescence emission with a quantum yield of 12% and inhibited the Gram-negative *E. coli* and Gram-positive *S. aureus* at a minimum inhibitory concentration (MIC) of 1 and 0.75 mg/mL, respectively. N-CDs were also used as nanocarrier for ciprofloxacin that was used as a model drug and its release was proven time-dependent. The release kinetics followed Korsmeyer-Peppas model at the physiological pH. Also, trinitrophenol (TNP), an explosive, was analyzed by using N-CDs via fluorescence quenching (Saravanan et al., 2020). Zhao et al. (2019), developed nitrogen and phosphorus co-doped CDs (N, P-CDs), which were used as effective antibacterial material against *S. aureus* and *E. coli*. The developed CDs were also utilized for fluorescence imaging of *S. aureus*, and as a fluorescence probe for the detection of Sudan Red I (Zhao et al., 2019). Demirci et al. (2020), prepared N-CDs using PEI and citric acid (CA) as starting material. The N-CDs were prepared at a yield of 53% ± 4.8% as per quinine sulfate standard using PEI:CA in 1:1 wt. ratio. These N-CDs inhibited the development of both Gram-negative and Gram-positive bacteria without any toxicity to *in vitro* mammalian cells. Additionally, skin permeation studies indicated good permeation of CDs into and through the dermis, showing their topical administration potential (Demirci et al., 2020). Koulivand et al. (2020), prepared N-CDs added to polyethersulfone (PES) to create new antifouling and antibacterial nanofiltration membranes using hydrothermal technique. All the N-CD-blended membranes displayed improved performance compared to the plain PES when evaluated. It also showed a flux recovery ratio (*FRR*) of 73.1% revealing its antifouling properties. Membrane showed inhibition of both Gram-positive and Gram-negative bacteria in antibacterial assessment showing antibacterial potential of N-CD-blended membranes (Koulivand et al., 2020).

John et al. (2020), developed photoluminescent CDs from wheat bran using hydrothermal method in which they conjugated CDs with amoxicillin (AMX) in order to assess the application of CDs for delivery of antimicrobial drug. CDs showed blue-green fluorescence emission and higher drug loading efficacy of 69.8% particularly in CD-AMX: 2-800. CD-AMX release profile was tested in different pH conditions (5, 6.8, and 7.2) and showed initial rapid release followed by sustained release up to 70 h. The CD-AMX conjugate showed faster release in acidic conditions (pH-5.0) due to protonation of AMX β-lactam ring in acidic conditions, and thus showed that the conjugate CD-AMX confer sustained pH-sensitive drug delivery. MTT assay was performed at various dilution of CD-AMX prepared using ratio of CD-AMX:2-400 and results showed higher survival rate of 92.23% using 25% diluted CD-AMX. Prepared CDs showed effectiveness against Gram- negative and Gram- positive bacteria with causing significant toxicity, showing its potential as a promising carrier for antibacterial drug delivery (John et al., 2020). Antimicrobial photodynamic therapy (PDT) is an alternate therapeutic technique which is cost-effective and practicable. For this objective, octa-imine substituted zinc phthalocyanine (ZnPc) was synthesised and attached to nitrogen, sulphur co-doped graphene quantum dots (N, S-GQDs) through π-π stacking. Prior to *in vitro* cell studies, the photochemical and photophysical properties of Pc alone and Pc-conjugated to the GQD nanomaterial were investigated in solutions, including fluorescence, absorption, fluorescence lifetime, singlet oxygen quantum yields, triplet state quantum yields, and excited state lifetimes. Results showed that photodynamic inactivation increased with ZnPc conjugation to N, S-GQDs (Sen and Nyokong, 2021).

#### 3.1.2 CDs-mediated delivery of antimicrobials

Romero et al. (2021), studied antimicrobial photodynamic effect of CDs developed from citric acid against *S. aureus* suspension and biofilm. *In vivo* studies were conducted in mice with wounds contaminated with *S. aureus.* The viability test showed 10^4^ log reduction in bacterial count on the skin lesions via CDs-mediated photodynamic inactivation. These findings showed promising application of CDs based antibacterial PDT in the treatment of wounds infected with Gram-positive bacteria (Romero et al., 2021). Das et al. (2020), prepared fluorescent surface-quaternized CDs (JB-CDs) sonochemically from jute caddies and modified their surface characteristics by adding benzalkonium chloride (BZC). Results showed that JB-CDs exhibited good water solubility, photostability and excitation-dependent emission. These JB-CDs were used as a fluorescent nano switch for the detection of inorganic pollutants in aqueous solutions, such as chromium (VI) [Cr (VI)] ions. To confirm antibacterial activity, JB-CDs were tested against *S. aureus* and *E. coli* indicating substantial inhibition of bacterial growth. Apart from this, JB-CDs were studied as nanocarrier for drug delivery of ciprofloxacin. *In vitro* release study results showed pH-responsive controlled release behavior of JB-CDs at physiological pH (Das et al., 2020). In another study, Liang et al. (2021), reported antibacterial activity of CDs incorporated with levofloxacin hydrochloride. CDs showed capable antibacterial properties against both Gram-positive and Gram-negative bacteria without causing any cytotoxicity. Mechanism of antibacterial action of CDs included their physical/chemical attachment to cell membrane, surface wrapping and then cell membrane breakdown, which caused increased production of reactive oxygen species (ROS) within the cell without the use of additional light or oxidant (Liang et al., 2021). Saravanan et al. (2021), prepared CDs from *Curcuma longa*, which were examined for their antibacterial efficacy against Gram-positive bacteria, *S. aureus* and *S. epidermidis*, and Gram-negative bacteria, *E. coli* and *K. pneumoniae*. Sun et al. (2021), performed a study in which chlorhexidine gluconate-derived CDs were divided into three groups on the basis of their particle size by applying different molecular weight cut-off membranes. Their findings revealed that antibacterial activity against Gram-negative bacteria and Gram-positive bacteria improves as CDs size decreases. This phenomenon might be caused by changes in the cellular absorption and CDs distribution in the cell membrane, or by a limitation between the polar functional group and the DNA molecule (Sun et al., 2021). Zhao et al. (2021), synthesized CDs with blue-green fluorescence under low reaction temperature (30 min, 60°C). The inhibitory action of developed CDs against diverse microbes, including bacteria and fungus, were compared using inhibition zone tests and minimum inhibitory concentration (MIC) studies (Zhao et al., 2021). In another study, negative charge CDs were prepared from citric acid and urea as precursors and evaluated for their antibacterial action against MDR bacteria. Results revealed that the developed CDs have the potential to be effective against MDR *S. aureus* and can be used as an alternative to antibacterial treatment (Kung et al., 2020). Boobalan et al. (2020), developed fluorescent blue/green CDs using oyster mushroom and demonstrated their antibacterial activity. Ye et al. (2020), developed CDs for antibacterial action against *S. aureus* and *E. coli* using *p*-phenylenediamine as carbon source through a simple hydrothermal method. The minimum bactericidal concentrations of the CDs were found to be 2 and 30 μg/mL, against *S. aureus* and *E. coli*, respectively (Ye et al., 2020). Similarly, Pandey et al. (2021), prepared CDs from citric acid and β-alanine. Its antimicrobial activity was studied against several Gram-negative bacteria, including *E. coli*, *Salmonella*, *Agrobacterium*, *Pseudomonas*, and *Pectobacterium* species. Results from this showed that CDs can act as an effective alternate to commercially available antibiotics (Pandey et al., 2021). Ahuja et al. (2021), synthesized CDs using xylitol (XCDs) which were conjugated with antimicrobial drugs ketoconazole and tetracycline for fungi and bacteria, respectively. Results showed higher inhibitory potential with reduced dose of antimicrobials using XCDs as compared to xylitol against *C. neoformans, C. albicans, S. pyogenes*, *E. coli*, *L. monocytogenes* and *S. typhi.* Drug delivery was also improved in the presence of XCDs showing drug carrier potential of CDs (Ahuja et al., 2021). Levofloxacin-based CDs (LCDs) with low drug resistance and greater antibacterial activities were developed by Wu et al. (2022). *In vitro* and *in vivo* studies showed excellent antibacterial activity of LCDs (Wu et al., 2022). The antibacterial activity of recently reported CDs and their applications in delivery of antimicrobials are summarized in Table 1.

More research in the use of CDs as antimicrobials can be explored by preventing ROS from damaging the normal cells. To further improvise the antimicrobial efficacy, it is needed to investigate the use of high performing precursor as a source of carbon for the synthesis of CDs and that should be water soluble and dispersible for ease of penetration and delivery.

### 3.2 CDs in ocular drug delivery

Ocular infection such as bacterial keratitis or endophthalmitis are not uncommon and various nanomaterials such as silver, copper oxide, iron oxide, titanium oxide, and zinc oxide particles received attention for their anti-bacterial properties. However, the toxicity of these inorganic materials and metal oxide poses a major concern and can be circumvented with the use of CDs. Moreover, the small nanoparticle size of CDs (<10 nm) further makes them suitable drug delivery vehicle as it causes no or minimal irritation in the eyes following administration (Garner et al., 2020).

Though CDs have good potential in novel biomedical applications due to their physicochemical properties like biocompatibility, fluorescence, and conjugation ability with various therapeutic agents, there are limited studies available regarding their applications for ocular drug delivery (Garner et al., 2020). Jian et al. (2017), developed an antibacterial agent for topical treatment of bacterial keratitis (BK) using carbon quantum dots (CQDs). The possible antibacterial mechanism suggested that the super-cationic CQDs prepared by direct pyrolysis of spermidine (Spd) (CQD_Spds_) had a small size and a large positive charge which ruptured the bacterial membrane. In addition, topical ocular application of CQD_Spds_ can cause the tight junction of corneal epithelial cells to open, resulting in a significant antibacterial effect in rabbits with *S. aureus* induced BK. These findings demonstrated CQD_Spds_ to be a viable antibacterial option for the treatment of eye-related bacterial infections and even chronic bacteria-induced illnesses (Jian et al., 2017). Shoval et al. (2019), developed ocular nanomedicine using hybrid aptamer modified-CDs for controlled release of inhibitors of vascular endothelial growth factor (VEGF), which plays a main role in the pathogenesis of angiogenic ocular diseases. The hybrid nanoparticles were made using CDs that had been functionalized with the VEGF aptamer. The hybrid CDs efficiently suppressed VEGF-stimulated angiogenesis in choroidal blood vessels in both *in vitro* and *in vivo* models (Shoval et al., 2019). Wang et al. (2022b), developed ocular drug delivery system based upon CDs embedded in thermosensitive *in situ* gel for the topical administration of diclofenac sodium (DS). *In vitro* results showed sustained release of DS for 12 h and *ex vivo* fluorescence delivery indicated that this system could be used for cell imaging and ocular tissues tracing. A positive charge on the composite of DS-CD along with the gel prolonged the precorneal retention and thus improved the bioavailability (Wang et al., 2022b). In another study, Wang et al. (2021a), prepared a composite system for ocular delivery that combined self-targeted CDs and thermosensitive *in situ* hydrogels. Electrostatic interactions were used to load DS onto the surface of CDs, resulting in DS-CDs nanoparticles that have the characteristics of biphasic drug release. The *in vivo* fluorescence and corneal penetrability studies revealed that drug retention duration was extended, and corneal transmittance was increased. The DS-CD Gel demonstrated high cytocompatibility and CD44 targeting in cellular studies (Wang et al., 2021a). There are only limited number of scientific reports available on CDs application in the ocular field, therefore the potential application of CDs in this area needs to be explored further.

### 3.3 CDs for drug delivery in the brain

The human brain is the most complex interconnected network and the most important part of the body. Major causes for brain disorders are illness, genetic changes and traumatic injury. Main hurdle for drug delivery in brain is the BBB (Dong, 2018). Drug delivery across the BBB is very challenging, particularly in brain tumor and Alzheimer’s disease (AD). Most remedies and drugs have limited application when it comes to brain delivery due to their inability to cross the BBB. To that end, some exciting research is being performed to enhance the delivery of therapeutic agents to the brain using nanomaterials, especially CDs. CDs were reported to cross the BBB through passive diffusion owing to their very small size, amphiphilicity, positive surface charge and receptor-mediated endocytosis (Zhang W. et al., 2021).

#### 3.3.1 CDs-mediated BBB permeability

CDs also have limitations regarding BBB penetrability as other small molecules. Mintz et al. (2019), proposed that if CDs can be synthesized from a precursor molecule that can cross the BBB, there are chances that the attached original precursor molecule with the CDs can enter the brain. As a result, they produced tryptophan CDs utilizing the amino acid method for crossing the BBB via LAT1 transporter-mediated endocytosis. Urea and 1,2-ethylenediamine were used as nitrogen source to develop tryptophan CDs. These CDs were evaluated in zebrafish central nervous system to assess their BBB permeability. Results revealed that CDs prepared with tryptophan amino acid acted as a promising carrier system for drug delivery and imaging in the brain (Mintz et al., 2019). Zhou et al. (2019), synthesized yellow-emissive CDs (Y-CDs) as promising drug nanocarriers across BBB using *o*-phenylenediamine and citric acid as raw material. The ability of synthesized Y-CDs to cross BBB was evaluated in wild-type zebrafish using confocal image analysis. Results showed that Y-CDs crossed the BBB most possibly by means of passive diffusion owing to their amphiphilic nature. Furthermore, Y-CDs suppressed the overexpression of human amyloid precursor protein (APP) and amyloid (A), both of which are involved in AD pathogenesis. Hence, results suggested the drug delivery potential of Y-CDs in brain as well as their ability to inhibit Aβ-related pathology in AD (Zhou et al., 2019). Zhou et al. (2020), directly conjugated two different CD models, black CDs (B-CDs) and gel-like CDs (G-CDs) for potential application of CDs as nanocarrier for brain delivery. As a result of conjugation, black-gel CDs (B-G CDs) possessed properties from both CDs, such as greater thermostability, better aqueous stability and red-shifted photoluminescence emission. Also, nanostructure formed with the decreased mass ratio of B-CDs to G-CDs showed good potential of CDs as Lego-like building blocks. Moreover, zebrafish bioimaging exhibited the bone targeting and BBB crossing potential of B-G CDs (Zhou et al., 2020). Despite showing promising drug carrier potential, a better understanding of drug loading capacity and release kinetics needs to be investigated. Few *in vitro* studies have shown CDs to produce an immune response at high concentrations (Lategan et al., 2018). Therefore, it is also important to optimize the dose of CDs for future clinical studies.

#### 3.3.2 CDs as nanocarriers for delivery of neuroprotective drugs

In order to treat a brain-related medical condition, delivery of therapeutic agent across BBB was a major bottleneck. Since CDs have the ability to cross BBB, various therapeutic agents can be delivered in desired concentration to achieve a desired pharmacological effect in the brain. Herein, we have discussed studies showing efficacy of CDs in delivering therapeutic agents either *in vitro* or *in vivo*. Hettiarachchi et al. (2019), developed CDs based triple conjugated system for delivering drug in glioblastoma brain tumors. Triple conjugated system of CDs was formed with targeted ligand transferrin and anticancer drugs, temozolomide and epirubicin. Glioblastoma brain tumor cell lines were used for *in vitro* evaluation. The efficacy of the synthesized triple conjugated CDs system was compared to free drug combinations, non-transferrin CDs–drugs, and dual conjugated systems (single drug conjugation along with transferrin). Triple conjugated CDs system at a very low concentration showed the lowest cell viability. Moreover, the triple conjugated CDs system caused more cytotoxicity to brain tumor cell lines as compared to other groups (Hettiarachchi et al., 2019). Chung et al. (2020), developed β-amyloid (Aβ) protein targeted photomodulating CDs for treatment of AD, as Aβ peptide aggregates worsen neuropathy and cognitive impairment in AD. Similarly, nanocarrier ability of carbon nitride dots (CNDs) were evaluated for targeted pediatric glioblastoma cells using gemcitabine as model drug by Liyanage et al. (2020). In another work, Li et al. (2021), created new boron-containing CDs (BCDs) for boron neutron capture therapy (BNCT) for glioma treatment with higher water solubility and excellent optical properties for tracing ^10^B *in vitro* and *in vivo*. Fluorescent imaging showed internalization of BCD–Exosomes around the nuclei of U-87-MG glioma cells *in vitro*. Prominent BNCT effect of the BCD–Exosomes-treated brain glioma in the mice model was demonstrated with 100% survival ratio (Li et al., 2021). CDs derived from metformin (Met-CDs) were developed for mitochondrial and nucleus localizations along with BBB penetration. Bioimaging studies showed Met-CDs penetration in the cell membrane and their localization specifically inside the cancer cells mitochondria. *In vivo* study performed in zebrafish study confirmed BBB penetrability of Met-CDs without the prerequisite of any other ligands (Kirbas Cilingir et al., 2021). Zhang et al. (2021c), derived novel *Crinis Carbonisatus* CDs (CrCi-CDs) obtained from carbonization of human hair, and investigated their neuroprotective effect against cerebral infarction in stroke. Neuroprotective study results in *in vivo* middle cerebral artery occlusion (MCAO) model showed significant reduction in neurological deficits (Zhang et al., 2021c). Presently, most CDs based nanocarriers showed BBB penetration ability which depends upon specific ligands for ligand-mediated endocytosis. So, self-targeting CDs with potential of penetrating the BBB need to be explored further by considering various precursors, drug and nanocarrier compared to non-self-targeting CDs. Apart from this, CDs mechanism for BBB penetration need to be understood well for their CNS drug delivery applicability (Zhang W. et al., 2021).

### 3.4 CDs as nanocarrier for gene delivery

Progress of novel multifunctional gene delivery systems with greater efficacy is important. In recent years, CDs has emerged as an imaging-trackable nanocarrier for gene delivery applications which may yield nanoparticles with positive charge for interaction with the nucleic acids bearing negative charge (Mickaël et al., 2019; Mohammadinejad et al., 2019). Due to their high transfection efficiency (TE), CDs can be used efficiently for plasmid DNA and siRNA delivery to the targeted cells without any toxic effects (Mohammadinejad et al., 2019). He et al. (2019), synthesized CDs from Gd(III) salts/complexes (L-CD/C-CD), cationic polymers, and citric acid for application in gene delivery and multi-modal (MR/FL) imaging. *In vitro* gene transfection studies using L-CD exhibited 74 times higher efficiency of transfection and anti-serum abilities compared to PEI 25 kDa. Gene delivery process was evaluated by confocal laser scanning microscopy, which showed blue or green fluorescence in HeLa cell lines. Besides, the L-CD/C-CDs possess appropriate particle size that resulted in increased buildup at the tumor location via the enhanced permeability and retention effect (EPR) thereby proving it to be more effective than a widely used contrast agent, i.e., Gd-DTPA (diethylenetriamine penta-acetic acid) in *in vivo* tumor-specific MRI imaging (He et al., 2019a). Another study from the same lab also reported two cationic polymer-derived CDs with blue fluorescence for creating biocompatible, multipurpose gene vectors with high TE (He et al., 2019b). Mickaël et al. (2019), developed cationic CDs by citric acid/bPEI600 (1/4, w/w) pyrolysis to remove the unreacted low molecular weight reagents which causes nanoparticles separation problem from residual polymer that is harmful to cells. Eleven CDs exhibiting greater water solubility were developed after evaluating reaction conditions and activation modes. CDs showed noticeable variation in their gene delivery efficiency in epithelial cell line A549 using a pDNA encoding the *Gaussia princeps* luciferase gene after having similar physical properties. CDs synthesized in domestic oven under microwave irradiation revealed to be superior to all the other CDs. It was also compared with the bPEI25k, gold standard transfection reagent which is associated with cytotoxicity. Results showed that optimal CD/pDNA w/w ratio shifted down to 2 while it was ca. 4 with bPEI25k and thus limiting the toxicity associated with bPEI and related compounds (Mickaël et al., 2019). Chen et al. (2020) *,* synthesized hydrophobically modified CDs from PEI via an epoxide ring-opening reaction. Study results presented the dual-channel imaging capability of CDs, due to which intracellularly delivered DNA can be tracked. Oleyl-modified-CDs showed 200 times more TE as compare to PEI 25 kDa in the presence of serum in A549 cells (Chen et al., 2020). In another study, CDs PAMAM nanohybrids were developed by self-assembling of CDs and G4-G6 (polyamidoamine) PAMAM-NH2 dendrimers for transfection and bioimaging purposes. The nanohybrids were found to be more photostable, compared to pristine CDs. These nanoscale hybrids were internalized in the cells with excellent TE thereby proving promising applications in the biomedical field (Martins et al., 2021). In summary, more studies are needed to fully understand the interaction of CDs with living cells and to further explore their potential in living cells or species that are resistant to transformation. More work needs to be pursued at the clinical level for efficient bench-to-bedside translation of CDs as a carrier for gene delivery.

### 3.5 CDs as nanocarrier in anticancer drug delivery

Cancer refers to a group of diseases which can affect any part of the body causing uncontrollable and abnormal cell growth which potentially invades or spreads to the adjoining parts of the body. Over 100 types of cancer forms are there which are affecting humans worldwide. As per World Health Organization, it is estimated that cancer-related deaths are expected to increase to around 13.1 million by the year 2030 (Stewart and Kleihues, 2003). Advancements of various nanotechnology tools is being used for the treatment of various deadly diseases including cancer (Montané et al., 2020). CDs belonging to the family of carbonaceous nanomaterials are identified as potential candidates in nanotheranostics for the treatment, bioimaging and early diagnosis of cancer due to their exclusive optical properties and intrinsic theranostic properties, which are summarized in Table 2 (Jia et al., 2020; Singh et al., 2021).

**FIGURE 3:**
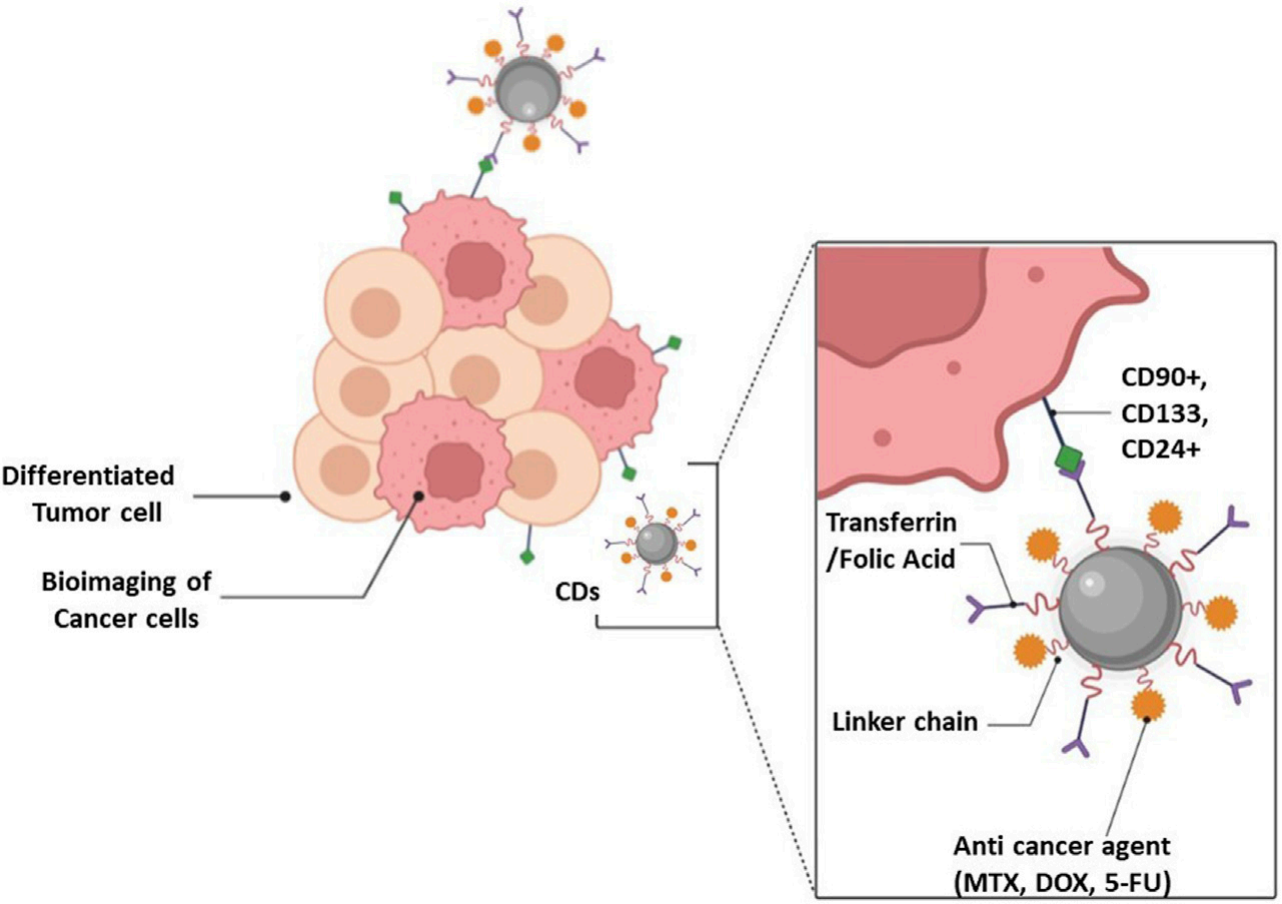
CDs theranostic application in cancer treatment.

Recent studies showed greater potential of CDs as nanocarriers in the field of cancer drug delivery and biological imaging. Das et al. (2019), developed sulfur and nitrogen doped photoluminescent CDs from κ-carrageenan and folic acid for cancer cell targeting. The folate receptor present on cancer cells led to remarkable cancer cells targetability by CDs which makes them a potential tool for biomedical studies as shown in Figure 3 (Das et al., 2019). Arsalani et al. (2019), developed polyethylene glycol passivated fluorescent CDs (CDs-PEG) from gelatin and PEG as nanocarrier for tumor treatment using methotrexate (MTX) as anticancer drug. The prepared CDs-PEG emitted blue photoluminescence with a maximum quantum yield of 34%. The effect of PEG on PL intensity of CDs was investigated and results showed stronger PL behavior of CDs-PEG as compared to pure CDs from gelatin. *In vitro* anticancer activity of CDs-PEG showed good antitumor efficacy than free MTX because of its greater *in vitro* nuclear delivery and thus proving the potential of CDs for targeted cancer therapy (Arsalani et al., 2019). Li et al. (2020a), developed smart mesoporous silica nanoparticles (MSNs) grafted with CDs and poly (N-vinylcaprolactam) (PNVCL) as a mixed shell (CDs/PNVCL polymer grafted MSNs) that allowed real-time monitoring and pH-triggered anticancer drug release. Doxorubicin (DOX) was loaded with the prepared CDs as a model drug. The study results demonstrated the qualities of excellent nanocarriers, such as low toxicity, limiting early release in the bloodstream by “caps” of CDs, and collapse of PNVCL. The composite allowed burst release of anticancer drug at the tumor site due to the rupture of Schiff base bonds. Through MTT experiments it was also suggested that CDs/PNVCL polymer grafted MSNs killed cancer cells without affecting normal cells. The ability of CDs fluorescence to monitor medication release in real time was studied and the results revealed linear connection in cumulative release of DOX and the fluorescence change of CDs (Li et al., 2020a). On the other hand, Su et al. (2020), reported that red-emissive carbon quantum dots (CQD), can be targeted to nuclei of cancer cells and cancer stem cells. DOX loaded on the surface of CQD reduced the cell viability of HeLa cells to 21% in contrast to 50% when cells were exposed to free DOX. The study results showed remarkable killing impact of CDs on tumor cells and improved treatment effectiveness with no recurrence (Su W. et al., 2020).

Li et al. (2020b), developed fluorescent-based CQD with photosensitizer functionalized and chemo drug-loaded nanocarrier system in which CQD and 5-aminolevulinic acid (5-ALA) were coupled with a mono-(5-BOC-protected-glutamine-6-deoxy) b-cyclodextrin (CQD-Glub-CD) moiety, and then DOX was loaded into the 5-ALA-CQD-Glub-CD system. *In vitro* drug release pattern of DOX from the system was found to be pH-dependent with higher release at acidic pH 2.8. The photodynamic action of ROS production causes cell damage and morphological alterations in cells tested for breast cancer cell line (Li et al., 2020b). Hailing et al. (2020), developed fluorescent CDs loaded with DOX and passivated with PEI (CD-PEI-DOX) as a drug delivery system for treating cancer. Results showed prolonged drug release from the system with improved toxic effect on liver cancer cells (MHCC-97L and Hep3B cells) compared to free DOX (Hailing et al., 2020). Sun et al. (2020), developed DOX conjugated CDs (DOX-CDs) for improved intracellular drug delivery and cellular imaging. Uptake of DOX-CDs by cancer cells was seen in fluorescent cell labelling studies. Moreover, it was also observed that the DOX release from DOX-CDse was endo-lysosomal pH-dependent. MTT assay performed in ovarian cancer cell lines showed the enhanced cytotoxic potential of DOX-CDs. Additionally, in an animal imaging study, the CDs displayed bright fluorescent signal and low toxicity after administration for 7 and 21 days (Sun et al., 2020). Liu et al. (2021), developed endogenous NO-releasing carbon nanodots for gas therapy as cancer treatment. *In vitro* studies performed on human breast cancer cell line MCF-7, female gastric cancer cell line BGC-823, male lung cancer cell line A549 and female leukemic cell line K562 showed that carbon nanodots killed tumor cells without affecting normal cells. *In vivo* results also showed antitumor efficacy of carbon nanodots (Liu et al., 2021). Wang et al. (2020), developed a CDs-DOX delivery carrier and imaging probe for liver cancer targeted therapy with high fluorescence quantum yield (97%). Folic acid, as targeting component was used to modify CDs (FA-CDs) to which DOX was further loaded to form FA-CDs-DOX. Confocal microscope showed excellent capability of fluorescence imaging of developed system in liver cancer cells. *In vivo* imaging study showed stronger fluorescence intensity of FA-CDs-DOX which makes it easy to penetrate tumor tissue and skin. *In vivo* tumor inhibition studies indicated the higher targeting ability of FA-CDs-DOX as compare to free DOX, showing improved therapeutic effect. Cutrim et al. (2021), showed the nanofabrication of drug delivery system based on the self-assembly of the 5-Flurouracil (5-FU) onto the surface of CQD. The 5-FU-CQD nanoconjugate was less hazardous to normal cells than free 5-FU at equal concentrations, yet it had the same anticancer effect against a breast cancer cell line (MCF-7) as free 5-FU. The findings clearly suggested that the 5-FU-CQD nanoconjugate may improve cancer therapy effectiveness by lowering the toxicity of 5-FU to normal cells, reduce side effects as well that are intolerable for patients while retaining the therapeutic efficacy of 5-FU (Cutrim et al., 2021). In another study, Qin et al. (2021), reported fluorescent CQDs for *in vivo* bioimaging and targeted cancer therapy. Similarly, Ren et al. (2021), developed a red emissive polymer CDs-based nanocarrier system of coptisine, a poorly bioavailable drug, with integrated functionality for *in vivo* and *in vitro* simultaneous imaging and drug administration. Red emissive CDs serve many purposes in this nanocarrier system, including drug carrier, EPR effect, and simultaneous imaging. *In vitro* test demonstrated that polymer-based red emissive CDs might provide a prolonged drug release and improve coptisine effectiveness against cancer cells. The fluorescent CDs usefulness as a tumor-targeted drug delivery carrier of coptisine for cancer treatment was proven in an *in vivo* investigation (Ren et al., 2021). Lv et al. (2021), synthesized multi-functional CDs using folic acid as nitrogen source and for tumor targeting. Gallic acid was used as a carbon source as well as active antitumor moiety. *In vitro* cell imaging studies and *in vivo* antitumor studies performed on HeLa cell lines showed targeted imaging and antitumor abilities (Lv et al., 2021).

There are various phytochemicals which are reported for its anti-cancer activity, curcumin is one of them which has been reported effective against various cancers but due to its low aqueous solubility, stability, and poor bioavailability, its clinical results were not satisfactory. Sharma et al. (2021), developed curcumin CDs (CurCDs) using curcumin as carbon source and as an active molecule owing to its anti-cancer properties. CurCDs were compared with the plain curcumin and evaluated further for its anti-proliferative, apoptotic, and anti-migratory activities in glioblastoma (GBM) cells. Study revealed the superiority of CurCDs over free curcumin in terms of improved stability and enhanced bioavailability (Sharma et al., 2021). Mohammadi et al. (2021), developed CDs-chitosan nanocomposite hydrogels for multicolor imaging of MCF-7 cancer cells. These nanocomposite detects *microRNA-21* in MCF-7 cancer cells and thus demonstrate enhanced sensing properties for biomarker detection (Mohammadi et al., 2021). 6-mercaptopurine (6-MP) used for treatment of acute lymphoblastic leukemia, suffers from short half-life, poor bioavailability with severe side effects. To overcome all these shortcomings, 6-MP was conjugated with CDs through GSH-sensitive carbonyl vinyl sulfide linkage, and biotin was attached to the surface. Resulting compound revealed excellent stability in the phosphate buffer saline (PBS), lower cellular toxicity on normal cell lines (CHO) and good activity, comparable to free 6-MP against MCF-7 and HepG2 cancer cells (Talib and Mohammed, 2021). In another study, folic acid-based CDs (FACDs) were investigated for DOX delivery and diagnosis by Fahmi et al. (2021). Wen et al. (2020), synthesized novel nitrogen and sulfur co-doped CDs with bright orange fluorescence for mitoxantrone (MTO) delivery. In wide pH range, the prepared CDs displayed good fluorescence stability in a wide range of pH and its fluorescence quenched by folate, showing its sensitivity to FA with a detection limit as low as 0.85 nM. CDs also exhibited biocompatibility and greater photobleaching resistance due to which they could be applicable in cell imaging and drug tracking. *In vitro* studies were performed using MTO-CDs and their results showed cell inhibition confirmed by MTT assay and change in cell morphology. The combination of MTO-CDs provide a nanosystem allowing simultaneous imaging and anticancer effect and the presence of fluorescence did not affect the cell killing property of MTO (Wen et al., 2020).

CDs are also reported to have potential application for photodynamic and photothermal therapy in diagnosing and treating cancer (Nocito et al., 2021). Metal and heteroatom-doped CDs have potential applications in PDT for cancer diagnosis and treatment (Sekar et al., 2022). Nasrin et al. (2020), synthesized nucleus targeting CDs for PDT in oral cancer treatment. Li et al., (2020c), prepared sulphur doped CDs (S-CDs), as an PI3/Akt pathway inhibitor thereby significantly reducing cancer cell survival with high PDT performance. In another study, nuclei acid targeted CDs were developed by Xu et al. (2021), from quinolone derivatives (Cl-CDs, I-CDs). *In vitro*, these CDs produces RNA fluorescence and due to iodine doping possesses PDT led to the killing of cancer cells (Xu et al., 2021). Chen et al. (2021b), fabricated an injectable hydrogel by developing amido modified CDs (NCDs) for simultaneous phototherapy and PDT for cancer treatment. Phototherapy agents were incorporated into hydrogels with high loading efficiency. NCDs, acting as phototherapy agent, showed effective tumor inhibition and thus provided a new strategy for advanced tumor treatment (Chen et al., 2021b). In another study, Yue et al. (2021), synthesized green fluorescent riboflavin-based CDs having singlet oxygen generation ability for PDT in cancer treatment. *In vivo* results showed significant inhibition of tumor growth after PDT with CDs (Yue et al., 2021). Novel iodine doped CDs (I-CDs) exhibiting PDT performance and nucleic acid targeting properties were synthesized by Xu et al. (2021). *In vitro* results revealed that photodynamic I-CDs significantly killed cancer cells thereby confirming the targeting capabilities of CDs (Xu et al., 2021). Hu et al. (2021), synthesized and evaluated Sn nanocluster CDs for potential application in PDT in cancer treatment. *In vitro* and *in vivo* study results showed promising potential of CDs in PDT (Hu et al., 2021). Also, novel green fluorescent fluorine and nitrogen co-doped carbon dots (F, NCDs) for image guided PDT of cancers were synthesized and evaluated by Wu et al. (2022). *In vitro* study on HepG2 cell lines showed higher photodynamic efficiency and better cell imaging capability of the F, NCDs (Wu et al., 2022). Conclusively, recently reported studies showed significant potential of CDs as photosensitizer in photodynamic and photothermal therapy in cancer treatment. Although the applications of CDs in cancer theranostics showed promising potential, various challenges limit its use in humans due to lack of information on its genotoxic potential. This needs to be investigated thoroughly so that the CDs can be delivered to the cell nuclei targeting bad genetic material in order to augment antitumor efficacy. Furthermore, currently phototherapy entails an imaging agent that has an overlapping absorption coefficient and lies in the biological transparency window of 650–950 nm. Therefore, it is an urgent requirement to develop CDs with emission in the NIR region.

### 3.6 CDs in controlled release and smart stimuli-responsive drug delivery system

**FIGURE 4:**
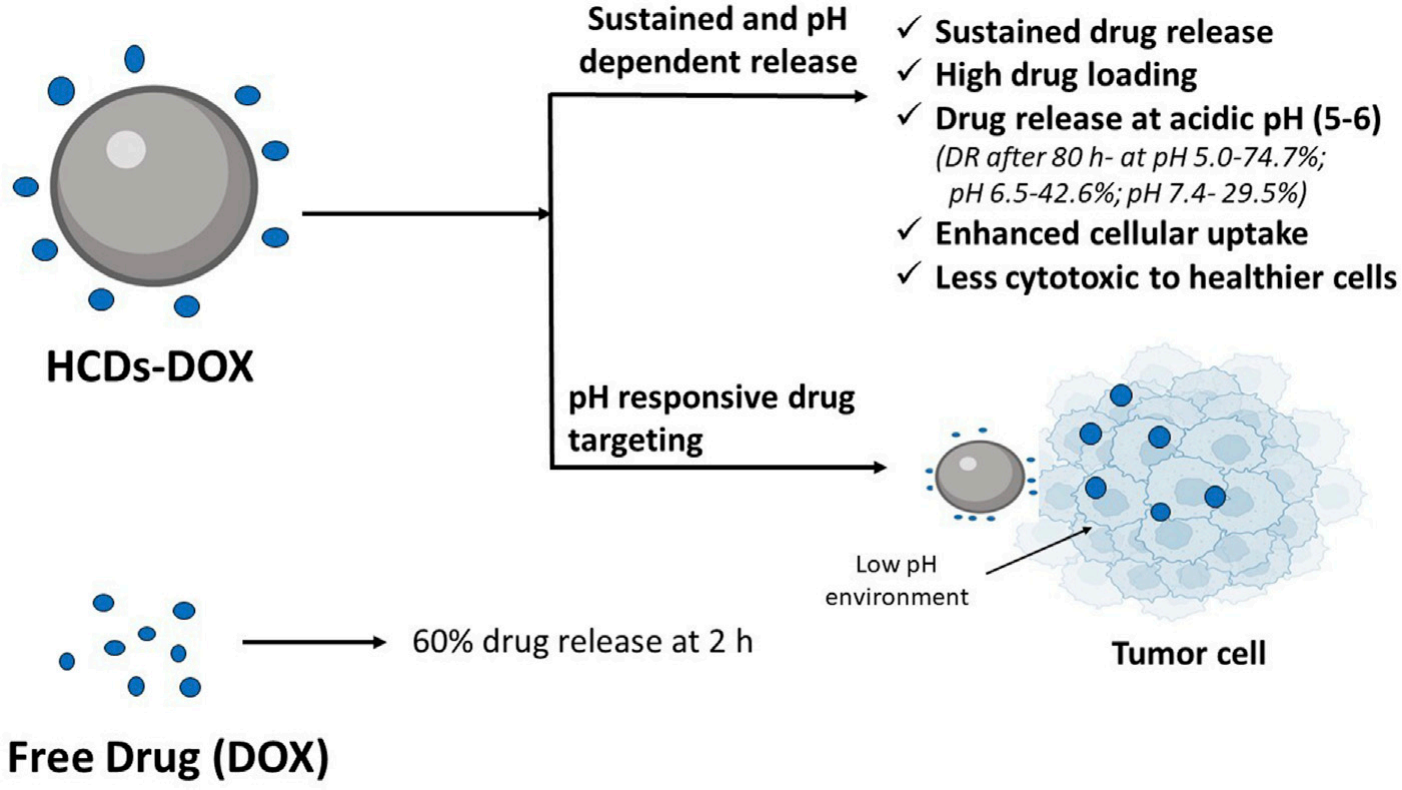
Hollow CDs (HCDs) for sustained and pH responsive drug delivery of doxorubicin (DOX). *In vitro* drug release study showed 74.7% of DOX release from HCDs at pH 5.0, 42.6% at pH 6.5% and 29.5% at pH 7.4 after 80 h. In case of free DOX release profile, 60% of the drug was released after 2 h (Zhang et al., 2019a).

A sustained drug delivery system tries to release the drug slowly for an extended period of time to maintain the constant concentration of the drug in blood or target tissue and thus leads to desired therapeutic effect in diseased conditions that requires drug concentration to be maintained for a long period of time. Smart stimuli-responsive drug delivery system uses natural or synthetic polymers that are designed to show therapeutic responses against the physicochemical and physiological processes, as well as external stimuli. This is particularly advantageous in reducing the side effects due to premature release of drug and assist in achieving the delivery of drug at the targeted site (Indermun et al., 2018). Zhang et al. (2019a), developed hollow CDs (HCDs) as a drug carrier for sustained delivery of DOX. After 80 h of *in vitro* drug release study, 74.7% DOX was released at pH 5.0, 42.6% at pH 6.5% and 29.5% at pH 7.4 (Figure 4). This was due to the dissociation of carboxylic acid hydrazine bond of HCDs-DOX by hydrolysis in acidic condition. In the case of free DOX release profile, no significant difference was observed at 5.0, 6.5, and 7.4 pH. 60% of the drug was released within 2 h. This indicated that free DOX could not reach the tumor cell effectively and HCD-DOX could be used as a potential sustained release drug delivery system for cancer treatment. The results of the study further demonstrated that HCDs-DOX effectively inhibited cancer cell proliferation and showed lower cytotoxicity to healthy cells as compared to DOX (Zhang et al., 2019a). Duan et al. (2020) introduced heparin (Hep), an anticoagulant, in conjunction with a drug delivery method based on CDs, i.e., DOX-CDs-Hyaluronic acid (HA) that can delivers DOX to cancer patients with the complication of thrombosis with reduced adverse effect. Drug release studies showed double trigger release of DOX from CDs-HA-Hep/DOX system due to acidic environment of the tumor and presence of HA, which was found to be dependent on the concentration of HA and pH value. *In vitro* MTT and scratch tests results further confirmed the inhibition of growth and migration of cancer cells, respectively. Visual tracking of the drug from the developed delivery system based upon CDs was also feasible (Duan et al., 2020). Chung et al. (2022), synthesized CDs/hydroxyapatite (CD-HAP) nanocomposite as a carrier system for acetaminophen using sugarcane bagasse char as a biowaste precursor via hydrothermal method. The pairing of CDs with hydroxyapatite improved the fluorescence property. The highest drug loading capacity of 48.5% was found in CD-HAP-40, which was synthesized using 40 mL of CDs. However, the best sustained acetaminophen release behavior was seen in CD-HAP-20 synthesized using 20 mL of CD, and the release kinetics followed Higuchi model showing diffusion mechanism of drug release. This study concluded the use of CD-HAP as a potential candidate for drug delivery application (Chung et al., 2020). Rahmani et al. (2021), developed pH-sensitive chitosan nanogel of rivastigmine by incorporating N-CDs. These nanogels exhibited pH-sensitive drug release. Swelling capacity and drug entrapment efficiency also improved by incorporating N-CDs in the nanogel. Many stimuli sensitive polymers exist that show immense potential, but their use is restricted due to their biocompatibility and toxicity related issues and therefore more research is warranted for safe and effective drug delivery using stimuli polymers.

### 3.7 CDs in vaccine delivery

CDs finds application in field of vaccine delivery and especially in cancer immunotherapy. The performance of therapeutic vaccine in cancer immunotherapy is not promising as it is not able to generate a robust immune response in body’s incapacitated immune system. The tumor associated antigen (TAAs) used in therapeutic vaccine is not able to stimulate the immune response resulting in limited success in developing vaccine for cancer. Ideally, a vaccine drug delivery system should be able to deliver antigen effectively and it should also act as adjuvant. Photoluminescent CDs are reported to have potential application as a vaccine adjuvant. Luo et al. (2018), successfully designed a nanocomposite consisting of uniform-sized CDs and tumor model antigen protein, ovalbumin (OVA). OVA can stimulate the maturation of dendritic cells resulting in augmenting the expression of costimulatory molecules, CD80 and CD86. It further led to the activation of T cells and subsequently their proliferation, vital for killing the cancer cells (Luo et al., 2018). Li et al. (2018), evaluated the efficacy of PEI modified fluorescent CDs as vaccine delivery system via intranasal route. In this study, a model protein antigen, i.e., OVA was delivered through intranasal route using PEI modified CDs as a carrier. Their results showed elevated levels of IgG and IgA titres and increased cytokine IFN-γ secretion by splenocytes, and memory T cells following administration. This study further validated the role of CDs as a carrier of vaccine antigen as well as also helps on trafficking the movement from the administration site to immune organs (Li et al., 2018). In another study, Huang et al. (2020), studied the role of quarternized cationic CDs prepared using biquarternary ammonium salt (BQAS-CDs) as a potent vaccine adjuvant *in vivo* and a viable alternative to alum in producing both Th1 and Th2 response. It is well known that alum is incapable of producing cellular immune response required for cancer management and microbial infections. In this study, OVA was used as a model antigen that is negatively charged and was adsorbed on cationic BQAS-CDs to form nanocomplex. *In vivo* studies in mice showed that the nanocomplex triggered Th2 humoral response which was measured by the IgG1 antibodies and was found to be similar to alum. However, the Th1 cellular immune response determined by measuring the IgG2a and IgG2b antibodies was more dominant in group of mice administered with BQAS-CDs + OVA nanocomplex. Furthermore, it was also found that a BQAS-CDs + OVA nanocomplex induce proliferation of OVA-specific CD4^+^ and CD8^+^ T cells compared to alum-OVA and naked OVA groups without affecting the hematological and histological parameters. Notably, the results of this study confirmed that BQAS-CDs can serve as a potential adjuvant and a delivery vehicle of antigen (Huang et al., 2020). Cheng et al. (2019), investigated the potential of CQD’s as a nanovehicle for protein antigen gp85. The protein antigen had been found to be effective in producing immune response against avian leukosis virus subgroup J (ALV-J) in chickens. Their study results revealed that the nanocomplex of gp85 with CQD produced high IgG levels and confer long-term protection. The study also confirmed the role of CQD’s as an adjuvant in potentiating the immune response (Cheng et al., 2019). Conclusively, several reports highlight the potential application of CDs as a nanovehicle for vaccine delivery. Furthermore, CDs-mediated vaccine drug delivery system needs to be explored preclinically and clinically to investigate the ability of CDs to induce cellular or humoral immunity.

### 3.8 Antiviral properties of CDs and as nanocarrier in antiviral drug delivery

The current approach of dealing with the viral infection is to prevent its adherence to host cells or to interfere with its replication. Virus eradication is an enduring challenge now a days and CDs play a vital role due to their antiviral properties. One of the studies conducted by Fahmi et al. (2016), demonstrated the potential of boronic acid modified CQD’s against HIV virus using MOLT-4 cells as a host cell system. The results of this study found that the boronic acid sites on CQD’s could bind to the glycoprotein expressed on HIV virus which is responsible for its adherence to host cells (Fahmi et al., 2016). A similar study was performed on CDs derived from benzoxazine monomer and found to be effective against a variety of viruses such as adenovirus-associated virus, porcine parvovirus, zika virus, dengue virus, and Japanese encephalitis virus. CDs hinders virus attachment to the host cells through the mechanism of binding to proteins present on the virus surface (Kotta et al., 2020).

There are studies that further explore the potential of modified CDs and found that it does not only interfere with the entry of viruses into host cells, but also affects its replication. Garg et al. (2020), found that the antiviral properties of CDs inhibiting the replication of virus can be achieved by doping it with heteroatom. In their study, CDs were doped with triazole derivatives and formed a series of bioisosters. The study results found that the heteroatom co-doped with CQD’s act by targeting the viral enzymes such as helicase and 3CLpro, required for viral replication. Triazaole derivatized CQD’s can be used to combat the SARS-CoV-2 infection, owing to the ability of virus to encode for non-structural proteins including the 3CLpro. Their mode of action is contrary to surface functionalized CQD’s that prevents the entry of virus to host cells (Garg et al., 2020). In another study, Łoczechin et al. (2019), studied the CQD’s potential to inhibit human coronavirus HCoV-229E. CQDs were linked separately with different chemical moieties to form seven different CQD’s and it was found that all of them inhibited the interaction of S protein receptor of HCoV-229E with the host cells. Chemical modification of CQD’s was done by introducing the boronic acid sites on it which resulted in augmentation of its antiviral property by interfering with S protein receptor and thus suppressed the syncytium formation. Preventing the syncytium formation led to prevention of HCoV-229E entry into the host cells. The timing of addition of CQD’s had been found to affect its mechanism of action as the addition of CQD’s after 5.5 h after the virus entered in host cells resulted in inhibition of genomic replication of virus. This could be due to interaction of CQD’s with the cell surface protein affecting the signal transduction and consequently replication or the interaction of CQD’s with the cytosolic proteins of cells after internalization. The antiviral effect of cationic curcumin CDs (CCM-CQD’s) was investigated, and it was found that they not only inhibit the viral entry but also its replication in a porcine epidemic diarrhea virus (PEDV) coronavirus model. The CCM-CQD’s interferes with the surface protein of virus and thereby preventing its entry into host cells. The study results also showed that CCM-CQD’s target viral RNA replication by producing interferon-stimulating genes (ISGs) and pro-inflammatory cytokines. Based on the study results, it was deduced that CCM-CQD’s can be used as alternative treatment strategy for patients infected with coronavirus (Ting et al., 2020).

Aung et al. (2020), developed amino phenylboronic acid-modified CQD’s (APBA-CQD’s) and conjugated it with antiviral drug, Duviral to further enhance its antiviral efficacy against HIV-1 infection. The modification on CDs improved its toxicity profile, biocompatibility, and imparts higher stability. The APBA-CQD’s acts by preventing the entry of virus to host cells by engaging with gp120 expressed on virus and thus prevents the formation of syncytia. Combination of APBA-CQD’s with Duviral, which is a multicomponent drug consisting of Lamivudine and Zidovudine, act by inhibiting the enzyme nucleoside reverse transcriptase and inactivate HIV-1 by intracellular and extracellular blocking. This combination showed better results in targeting virus and thus warrants further investigation as it is expected to improve the survival rate and life expectancy of patients (Aung et al., 2020). Iannazzo et al. (2018), also explored the potential of polycarboxylated graphene quantum dots (GQD’s) as a carrier of two antiviral drugs. The drug molecules chosen for the study were CHI499 and CDF119, which belong to the class of non-nucleoside reverse transcriptase inhibitors (NNRTI) and anchored to GQD’s. Results showed that the conjugate of GQD’s and antiviral drugs showed marked enhancement in antiviral efficacy compared to drug alone. The increased anti-HIV activity of the conjugate was due to dual nature of mechanism achieved by the conjugate. The NNRTI acts by inhibiting the reverse transcriptase and GQD’s acts by inhibiting the binding of virus to host cells (Iannazzo et al., 2018). In another study, Ju et al. (2020), successfully demonstrated the CQD’s-mediated delivery of locked nucleic acid (LNA) against Kaposi’s sarcoma-associated herpesvirus (KSHV) and AIDS related non-Hodgkin B-cell lymphoma. *In vitro* results showed that the conjugate of CQD-LNA acts by inhibition of proliferation of KSHV-associated primary effusion lymphoma (PEL) cells by inducing apoptosis and knockdown of KSHV micro-RNA’s, such as *miR-K12-1*, *miR-K12-4* and *miR-K12-11*. The conjugate had proven its efficacy in PEL nurtured xenograft mouse model by causing the regression of tumor growth and thus greatly increasing the survival rate (Ju et al., 2020). On similar lines, a study used antiviral compound glycyrrhizic acid (Gly), a traditional Chinese herbal medicine, as a precursor to synthesize antiviral CQD’s. It was found that Gly-CQD’s inhibited virus proliferation by approximately 5 orders of magnitude. This conjugate also provides the advantage of reduced side effects of glycyrrhizic acid that are more pronounced when given alone. Gly-CQD conjugate had been found to be act in many ways, namely: a) inhibiting virus proliferation; b) inhibiting virus invasion and replication; c) controlling the expression of antiviral genes; d) stimulating the production of interferons; e) regulating the production of virus-induced reactive oxygen species (ROS) (Tong et al., 2020; Xue et al., 2022). Undoubtedly, CDs confer alternative option in dealing with viral infection, however, most of the studies were performed *in-vitro* and there is a need to do more studies *in vivo* to validate the efficacy of CDs in the delivery of antiviral drugs. Additionally, CDs must be hydrophilic and exhibit colloidal stability in physiological conditions. A profound understanding of the structure activity relationship in terms of size, shape, surface charge, etc., to increase the efficacy of CDs as carrier of antiviral agents is required as well. Moreover, more research in developing CDs is required to combat virus mutation and the emergence of new strains.

## 4 Diagnostic applications of CDs

As a captivating part of fluorescent carbon nanomaterials, CDs demonstrates significant potential in bioimaging and biosensing field owing to their small size, better aqueous stability, excellent photostability and biocompatibility (Kong et al., 2020).

### 4.1 Bioimaging

Bioimaging enables visualization of cell and its orgnanelles that allows better understanding of their structure and physiological functions. In the past decade, the use of green fluorescent protein (GFP) in biomedical research allowed monitoring of interaction among proteins and gene expression. However, poor photostability and weak fluorescence of GFP restricted its further widespread utility. The discovery of fluorescent CDs possessed unique properties such as multicolor emission with high QY, tunable optical properties, excellent photostability and dispersion properties. CD-based optical imaging utilizes optical contrast between the image and surrounding region and enables early detection, screening, and diagnosis of life threatening disease conditions. The imaging capabilities of CDs is either due to its unique optical property, i.e., intrinsic fluorescence or due to the presence of functionality in its core or surface (Wang B. et al., 2022).

Herein, we have discussed the research outcomes of the various studies conducted using fluorescent CD probes. Hua et al. (2019) prepared red emissive, nucleolus targeted Ni–pPCDs using p-phenylenediame (pPDA) and nickel ions (Ni^2+^) to achieve the maximum quantum yield (QY) with excellent photostability. *In vitro* and *in vivo* studies revealed the biocompatibility of Ni–pPCDs and their utilization for high-resolution imaging of cell nuclei and high-contrast imaging of tumor-bearing mice and zebrafish (Hua et al., 2019). Zhang et al. (2019b), prepared N-CDs, a kind of green and red emissive CDs, that were loaded with multifunctional Mn^2+^ complex-modified polydopamine (PDA) nanoparticles (NPs) for fluorescent imaging. PDA-N-CDs (Mn) NPs can provide contrast for magnetic resonance imaging (MR) due to the automated chelation of Mn^2+^ ions. It was possible to effectively show the use of PDA-N-CDs (Mn) NPs for fluorescence, photothermal, and MR *in vivo* imaging. Furthermore, the PDA-N-CDs (Mn) NPs exhibited outstanding biocompatibility and minimal biological toxicity, as evidenced by cytotoxicity testing, hemolytic testing, histological analysis of viscera sections, and blood biochemical analysis (Zhang et al., 2019b). Mintz et al. (2021) showed that CDs are capable of quantitatively detecting water in organic solvents. Also, transferrin conjugation enhanced the CDs biocompatibility. These were used for cellular imaging of neuroblastoma cell lines (Mintz et al., 2022). Ren et al. (2020) developed blue fluorescent N-CDs using microwave-assisted method for cell imaging and metronidazole detection. Kong et al. (2020) developed an effective nanosystem based on the DNA aptamer AS1411 modified CDs with PEI as connecting bridge for bioimaging. Confocal microscopy and flow cytometry displayed higher cellular uptake of the CDs-PEI-AS1411 in MCF-7 breast cancer cells as compared to non-cancerous L929 fibroblast cells, thereby showing very specific capacity to identify nucleolin-positive cells. Results showed the application of synthesized CDs-PEI-AS1411 nanosystem in cancer cell targeted imaging (Kong et al., 2020). Gudimella et al. (2021) reported a sand bath assisted method for the preparation of fluorescent CDs using peels of citrus fruit. Prepared CDs revealed biocompatibility, low toxicity and significant free radical scavenging activity. Folic acid was conjugated with the CDs to enhance its potential as biological labels for cellular imaging at multiple excitations (Gudimella et al., 2021). CDs with protein like structure were created with the combination of fluorescent blinking domains and RNA-binding motifs, allowing for better nucleolar ultrastructure imaging. The picture enables precise differentiation of distinct cells from the same cell type by extracting multidimensional information from the nucleolus. Furthermore, it was shown that this CD-depicted nucleolar ultrastructure can be used as a sensitive hallmark to recognize and distinguish subtle responses to several stressors, as well as to provide RNA-related information that has previously been unavailable using traditional immunofluorescence methods. This protein-mimicking CDs might be used to investigate nucleolar stress in cell diagnostics and therapies (He et al., 2021). Ermis et al. (2022) also reported red emissive biocompatible CDs (R-CDs) based fluorescent probe for live 3D bioimaging of U-87 microglia tumor spheroids.

CDs exhibiting fluorescence in NIR region (700–1700 nm) are also gaining interest because of there application as nanomaterials in bioimaging. They are reported to be highly efficient in photothermal therapy. CDs exhibiting fluorescence in NIR window has a distinct advantage over other photoluminescent agents in high‐contrast *in vivo* fluorescence imaging owing to its deeper tissue penetration, high resolution, lower tissue self‐absorption/scattering, and less autofluorescence (Wang T. et al., 2021; Zhao et al., 2023). Bao et al. (2018) developed sulfur and nitrogen codoped NIR CDs for *in vivo* fluorescence and photoacoustic imaging with high photothermal conversion efficiency of 59%. Ding et al. (2019) synthesised highly fluorescent CDs emitting near infrared from lemon juice for potential bioimaging application. QY was reported to be 31%. Liu et al. (2022) fabricated large conjugated perylene derivates for synthesising CDs which exhibited high contrast *in vivo* NIR fluorescence bioimaging in mice model. Zhao et al. (2023) demonstrated application of CDs with NIR-I (700–950 nm) emission and NIR-II (1000–1700 nm) absorption in multiple photon bioimaging and photothermal therapy. Multiphoton-induced NIR-I emission with quantum yield of 29% and NIR-II induced heat with photothermal conversion efficiency of 41.19% was reported (Zhao et al., 2023). Overall, CDs exhibiting photoluminescence in the NIR region are reported to be of potential application in the field of bioimaging. However, synthesis of CDs exhibiting fluorescence in NIR region with higher QY is a major challenge.

Imaging using CDs emitting blue/green light of shorter wavelength have limitation of background interference and poor penetration in the biological tissues. Therefore, it is desired to use CDs that can emit in the NIR region while enabling good penetration of light in the biological tissue with minimal photodamage. Various studies have been done on different cell lines, mice and zebra fish have reported excellent biocompatibility of CDs, but more studies needs to be done at clinical level to ensure the safety of CDs. To gain more confidence in the use of CDs at clinical level, studies related to mechanism of interaction of CDs with biological system warrants further exploration.

### 4.2 Biological sensing

Biological sensing refers to identification and measuring the analytes concentration based on properties such as photoluminescence and electrical conductivity. The CDs are endowed with both of them and upon interaction with analytes lead to change in the optical emission characteristics such as change in fluorescence intensity or colorimetric wavelength. Based on these properties, CDs are further classified into three categories, i.e., on-off, off-on, and fluorescence shift. The on-off category is also called fluorescence quenching and used for the detection of cations, anions, and small molecules. In this, upon interaction of CDs with metal ion, the electron are transferred from CDs to empty d-orbital of metal ions resulting in fluorescence quenching. In the case of off-on category, the CDs regain its fluorescence and reach back to emission state upon interaction with the analyte. And lastly fluorescence shift category detects analytes based on partial overlap of donor emission and acceptor absorption spectra (Wang B. et al., 2022).

Herein, we have discussed the snapshot of various studies conducted by researchers on biosensing analytes using CDs. Zhu et al. (2019), prepared titanium carbides (Ti_3_C_2_) nanosheets mixed with red-emitting CDs (RCDs), where Ti_3_C_2_ showed selective and effective fluorescent turn-on nano sensor for glucose detection. The nanosensor can also be used to monitor glucose by using H_2_O_2_ produced by the oxidation of glucose catalysed by glucose oxidase (Zhu et al., 2019). For calcium ions detection, novel fluorescent CDs were developed in which the surface of the CDs was modified by ethylene-bis (oxyethylenenitrilo) tetra acetic acid (EGTA). The CDs emit bright blue fluorescence, and the intensity of the fluorescence declines significantly as the calcium concentration increases. The findings proved that calcium ion detection by CDs is a static fluorescence quenching mechanism. Also, cytotoxicity and cellular imaging investigations have demonstrated nontoxic and biocompatibility profile of CDs (Yue et al., 2019). Yu et al. (2019), prepared N-CDs which were extremely dispersible in water and can be utilised as an optical probe for label-free detection of Fe^3+^ via a switched off change. Also, in the presence of pyrophosphate, such sensing nanoplatforms can be restored. This on-off-on technique is likely to open up new opportunities for producing efficient sensors in industrial settings (Yu et al., 2019). A selective and environmentally friendly fluorescence immunoassay approach based on N-CDs for the exposure of nuclear matrix protein 22 (NMP22, antigen), was developed by Othman et al. (2020). The immunocomplex on the carboxylated N-CDs caused fluorescence intensity quenching. NMP22 was effectively detected in human urine samples using this approach, with recoveries ranging from 96.50% to 103.61%. These findings give substantial evidence that N-CDs can be used as fluorescent labels in immunoassays. Geng et al. (2020), reported a lysosome-specific fluorescent CDs for ATP detection in acidic lysosomes with “off-on” changes of yellow fluorescence. These CDs were effectively used for real-time monitoring of lysosomal ATP shifting concentration caused due to drug stimulation (e.g., chloroquine, etoposide, and oligomycin) (Geng et al., 2020). Orange–red emissive CDs (OR-CDs) were developed by calcining carbon sources like 5-amino-1, 10-phenanthroline (Aphen), and salicylic acid (SA), which worked as a “turn on” type fluorescence probe for Cd^2+^ detection. OR-CDs showed potential to reduce cadmium poisoning in living organisms in addition to being employed for intracellular Cd^2+^ imaging (Xu et al., 2022). Chattopadhyay et al. (2021), prepared CDs from peptic digest of animal tissue, which is easily available, without using any other chemical reagent for surface functionalization. The utilisation of these CDs as a fluorescent nanoprobe for the detection of metal ions (Cu^2+^ and Hg^2+^) via chelation, radical scavenging, and cellular imaging has been examined. They also displayed fast cell exocytosis, indicating that they have a lot of potential for chelation treatment. For fluorescence-based cell imaging, they can also be utilised as an alternative to synthetic organic dyes (Chattopadhyay et al., 2021). Tang et al. (2021), developed CDs using black pepper as raw material and designed them as NIR ratiometric nanoprobe for ascorbic acid measurement based on off-on mode with good selectivity and sensitivity. The suggested technique has enormous potential to increase the applications of dual-emission CDs in biological disciplines (Tang et al., 2021). Rajendran et al. (2021), reported green fluorescent CDs with quantum yield of up to 61% for tetracycline detection and mitochondrial labelling in cancer cells. CDs derived from coconut husk with good quantum yield and biocompatibility were synthesized by Chauhan et al. (2022), for sensing amino acid, L-tyrosine. Mathad et al. (2022), developed β-Cyclodextrin (CD) anchored neem (Azadirachta indica) CDs for greater electrochemical sensing performance of lapatinib, an anticancer drug, via host-guest inclusion. The proposed sensor could be used for determining lapatinib in pharmaceutical formulations and urine samples (Mathad et al., 2022). Blue fluorescent CDs conjugated with thiomalic acid (TMA)-capped AgInS_2_ quantum dots were developed and evaluated for determination of ibandronic acid, a bisphosphonate pharmaceutical, by Castro et al. (2022). Sohail et al. (2022), investigated Zn-doped CDs-based bioanalytical probe for the determination of antioxidant activity index (AAI) as traditional techniques lack truncated strength, high interference effect in complex samples, photo-bleaching and stability. Tang et al. (2022), synthesised nitrogen-doped fluorescent carbon dots from grape peel biowaste for detection of baicalin, a flavonoid compound used for liver cancer treatment. Further advancement in technology led to rapid detection of analytes via smartphones due to their ease of portability and accessibility. Su et al. (2020) developed light sensing CDs and integrate with smartphones that allows rapid detection of 2,4-dichlorophenoxyacetic acid in real time. Currently, there is limited application of this technology and more work is needed in the area to expand this technology for the detection of analytes in other disease conditions. Recently reported diagnostic applications of CDs are summarized in Table 3.

**TABLE 3:** Diagnostic application of CDs.

No.	Diagnostic approach	CD type	Conjugate/drug/precursor	Method of preparation	Applications	Ref.
1	Bioimaging	Red emissive CDs	Nucleolus targeted Ni–pPCDs using p-phenylenediame (pPDA) and nickel ions (Ni^2+^)	Hydrothermal method; QY was 64.9%	High-resolution imaging of nucleus, high contrast imaging of tumor-bearing mice and zebrafish	Hua et al. (2019)
		Mn^2+^ complex-modified polydopamine and dual emissive CDs based nanoparticles	Polydopamine (PDA) and manganese ion (Mn^2+^)	Self-polymerization; QY was 4.5% and photothermal conversion efficiency was 28.2%	Used in fluorescence, photothermal, and magnetic resonance (MR) *in vivo* imaging.	Zhang et al. (2019b)
		Red-emissive CDs	Transferrin conjugation o-pheylenediamine (o-PDA) as precursor	Oligomerization reaction	Quantitatively detection of water in organic solvents; Cellular imaging of neuroblastoma cell lines	Mintz et al. (2022)
		Blue, fluorescent nitrogen-doped CDs (N-CDs)	L-tartaric acid and triethylenetetramine	Microwave-assisted method, QY was 31%	Cell imaging and metronidazole detection	Ren et al. (2020)
		DNA aptamer AS1411 modified CDs	polyethylenimine (PEI)	Hydrothermal method	Have great detection ability of nucleolin-positive cells	Kong et al. (2020)
		Fluorescent CDs using peel of citrus fruits	Citrus peel and folic acid	Sand bath assisted green synthesis; significant free radical scavenging activity (EC50: 4.7382 μg mL^-1^)	Cancer cell imaging	Gudimella et al. (2021)
		Red emissive CDs	Paraphenylenediamine and thiourea	One-pot water based hydrothermal method; QY was 13%	Used for live 3D bioimaging of tumor spheroids	Ermis et al. (2022)
2	Biological and chemical sensing	Fluorescent CDs	Ethylene-bis (oxyethylenenitrilo) tetra acetic acid (EGTA)	Hydrothermal method	Calcium ion detection with detection limit (LOD) of 0.38 µM	Yue et al. (2019)
		Nitrogen doped CDs (N-CDs)	Citric acid and urea	Hydrothermal method	Detection of nuclear matrix protein (NMP22) in immunoassays with detection limit of 0.047 ng/mL	Othman et al. (2020)
		Lysosome-specific fluorescent CDs	5-amino-1, 10-phenanthroline (Aphen), and salicylic acid (SA)	Calcination	Detection of Cd^2+^ with LOD 0.30 µM	Xu et al. (2022)
		Blue emissive CDs	Peptic digest of animal tissue	Hydrothermal method; QY was 61%	Detection of metal ions (Cu^2+^ and Hg^2+^); Alternative to synthetic organic dyes for cellular imaging	Chattopadhyay et al. (2021)
		NIR ratiometric fluorescence CDs	Black pepper	Solvothermal method; QY was 10.25%	Ascorbic acid measurement	Tang et al. (2021)
		Green, fluorescent CDs	Triphenylphosphonium (TPP)	One-pot carbonization synthesis technique; QY was found to 61%	Detection of tetracycline and mitochondrial labelling in cancer cells	Rajendran et al. (2021)
		CDs	Coconut husk	Thermal calcination	Sensing amino acid and L-tyrosine with LOD of 0.96 nM	Chauhan et al. (2022)
		Nitrogen-doped fluorescent CDs (PT-NCDs)	Grape peel	Solid phase thermal method	Detection of baicalin used for cancer treatment	Tang et al. (2022)

## 5 Miscellaneous applications of CDs

Some other miscellaneous applications of CDs are reported in the literature, such as Kandra and Bajpai (2020), reported that impregnation of CDs into chitosan film caused seven-fold reduction in its water absorption capacity. Moisture permeation abilities of the plain chitosan and CDs loaded chitosan film matrix was found to be 1758 and 956 g/m^2^/day, respectively. Additionally, for chitosan film, bovine serum albumin (BSA) adsorption was found to be 24.2 mg/m^2^ which got reduced to 14.1 mg/m^2^ for CDs loaded chitosan film matrix. (Kandra and Bajpai, 2020). Lee et al. (2020), developed a CDs nanoparticle to investigate their potential as a new antiplatelet agent for arterial thrombotic diseases treatment without causing any cytotoxicity. Antiplatelet action was exerted due to the inhibition of collagen-stimulated human platelet aggregation. *In vivo* study showed reduced survival in mice with ADP-induced acute pulmonary thromboembolism without altering the bleeding time, which is the foremost adverse effect of antiplatelet agents (Lee et al., 2020). Zhao et al. (2022), evaluated Armeniacae Semen Amarum (ASA)-Carbonisata-derived CDs (ASAC-CDs) for its anti-inflammatory effects in rat model of acute lung injury (ALI). ASAC-CDs reduced inflammation by reducing the level of inflammatory mediators. CDs also showed antioxidant activity by reducing malondialdehyde (MDA), myeloperoxidase (MPO) content and increasing superoxide dismutase (SOD) activity and glutathione (GSH) content. Results demonstrated the potential application of developed system in clinical anti-pneumonia (Zhao et al., 2022). Gudimella et al. (2022), prepared fluorescent CDs from Carica papaya leaves which were examined for free radical scavenging activity, antioxidant activity and *in vitro* anti-inflammatory activity.

## 6 Conclusion and future perspectives

CDs are emerging nanoparticles due to various advantages, such as high photostability, less cytotoxicity, biocompatibility, high chemical inertness, eco-friendliness, nontoxicity, easy functionalization, non-blinking photoluminescence, and good water solubility. CDs demonstrates potential drug delivery applications as nanomaterial for treating cancer, brain, ocular and infectious diseases. They also have application in gene, vaccine and antiviral drug delivery. Due to their versatile physicochemical properties, CDs are capable of achieving sustained release action, pH responsive drug delivery, smart stimuli-responsive and targeted drug delivery. Although CDs based nanomaterials are found to be promising nanocarriers in diagnostics and therapeutics drug delivery, further advances are still desirable to enhance their performance, sustainability, durability and cost-effectiveness for efficient industrial scale-up. Although, numerous cell lines and *in vivo* cytotoxicity studies have reported that the CDs based nanomaterials did not induce any toxic effects, more detailed investigations are still required to explain their toxicity profiles and biodegradation mechanism for efficient drug delivery application. CDs needs to be explored for preclinical long-term toxicity before proceeding to their clinical applicability.
